# Proteomic analysis of cellular soluble proteins from human bronchial smooth muscle cells by combining nondenaturing micro 2DE and quantitative LC‐MS/MS. 1. Preparation of more than 4000 native protein maps

**DOI:** 10.1002/elps.201400573

**Published:** 2015-06-17

**Authors:** Ya Jin, Jun Zhang, Qi Yuan, Takashi Manabe, Wen Tan

**Affiliations:** ^1^ School of Bioscience and Bioengineering South China University of Technology Guangzhou P. R. China; ^2^ Guangdong Provincial Key Laboratory of Fermentation and Enzyme Engineering South China University of Technology Guangzhou P. R. China; ^3^ Key Laboratory of Industrial Biotechnology of Guangdong Higher Education Institutes South China University of Technology Guangzhou P. R. China; ^4^ Faculty of Science Ehime University Matsuyama Japan

**Keywords:** Cellular proteins, LC‐MS/MS, Native protein map, Nondenaturing micro 2DE, Quantitative protein–protein interaction

## Abstract

Soluble proteins of human bronchial smooth muscle cells (HBSMC) were separated by nondenaturing micro 2DE and a 30 mm × 40 mm area of the CBB‐stained slab gel (1.0 mm thick) was cut into 1.1 mm × 1.1 mm squares, then the proteins in the 972 gel pieces (squares) were applied to quantitative LC‐MS/MS. Grid‐cutting of the gel was employed to; (i) ensure the total analysis of the proteins in the area, (ii) standardize the conditions of analysis by LC‐MS/MS, (iii) reconstruct the protein distribution patterns from the quantity data [1]. Totally 4323 proteins were identified in successfully analyzed 967 squares and the quantity distribution of each was reconstructed as a color density pattern (a native protein map). The quantity of the proteins distributed from 3.6% to 1 × 10^−5^% of the total protein quantity in the grid area. Each protein map was characterized by several features, including the position of quantity peak square, number of detected squares, and degree of concentration (focused or dispersed). About 4% of the proteins were detected in 100 or more squares, suggesting that they might be ubiquitous and interacting with other proteins. In contrast, many proteins showed more concentrated quantity distribution and the quantity peak positions of 565 proteins with a defined degree of concentration were summarized into a quantity peak map. These results for the first time visualized the distribution patterns of cellular proteins on a nondenaturing 2D gel.

**Abbreviations**
DPBS: Dulbecco's phosphate‐buffered saline
FBS: fetal bovine serum
HBSMC: human bronchial smooth muscle cells
HDL: high‐density lipoprotein
HDMS^E^: ion mobility separation‐enhanced MS/MS in data‐independent acquisition mode
IMS: ion mobility separation
LMW: low molecular weight
nano‐UPLC: nano‐ultra performance liquid chromatography

## 1 Introduction

In order to reconstruct the structure and function of complex protein systems, various methods of protein separation and analysis have been developed. Up to 1980s, the major aim of protein separation methods was the purification of proteins into single polypeptide level, since the structural analysis of proteins could be done mainly by de novo amino acid sequencing. 2DE run under denaturing conditions was designed aiming at the separation of all the cellular polypeptides into single spots on a slab gel 2. Technical difficulties in the structural analysis and assignment of minute quantities of polypeptides on the 2D gels were overcome in 1990s by the advent of mass spectrometric techniques that utilize the information in protein sequence databases. However, the improvements of MS apparatus and related software in peptide analysis, especially the high sensitivity and high throughput obtained by nano‐LC‐MS/MS systems, enabled the analysis of polypeptide components in complex protein systems without the extensive purification of the polypeptides by 2DE. Most typically, solubilized proteins were directly digested with proteases and the peptides were analyzed by two‐step HPLC followed by MS/MS to identify the proteins 3, 4, 5. Adding the step of gel filtration at the protein level before digestion with proteases and employing nano‐LC combined with a high‐resolution MS apparatus, about 10 000 proteins in a human cancer cell line 6 and in 11 mammalian cell lines 7 were identified. Directly digested peptides were cleaned by C18 reversed‐phase spin column, separated on pH 3–10 IPG strips, and employing nano‐LC combined with a high‐resolution MS apparatus, about 10 000 proteins were identified with quantity information 8. One‐dimensional SDS‐PAGE was used to separate proteins, entire gel lanes were sliced into 50 equal pieces, proteins were in‐gel digested, and subjected to reversed‐phase LC‐MS/MS and 2000–4000 proteins were identified in each of the seven human cell lines 9. The addition of the step of solution isoelectric focusing before 1D SDS‐PAGE was reported to improve the reproducibility of proteome coverage in a human cancer cell line 10. Therefore, the intended use of denaturing 2DE became to be more focused on comparative studies between several samples 11, 12 and on the studies of PTMs 13.

Nondenaturing methods of protein separation had disadvantages in structural analysis of the separated proteins, since one purified protein can be comprised from multiple polypeptides. However, the advent of mass spectrometric techniques mentioned above enabled structural analysis of proteins and protein complexes separated by nondenaturing methods. Blue‐native 2DE was combined with mass spectrometric techniques and applied for the analysis of protein–protein interactions in *E. coli* cytosol and membrane fractions 14. We have been using a technique of nondenaturing micro 2DE that can separate proteins maintaining their biological structures and functions, and combined it with MALDI‐MS‐PMF in analyzing *E. coli* cytosol proteins and protein complexes 15, 16. In the course of examining the performance of a quantitative LC‐MS/MS apparatus, we realized that its sensitivity in protein structural analysis exceeded the detection sensitivity of the conventional staining methods of proteins on nondenaturing micro 2D gels. Therefore, we grid‐cut an area (5 mm × 18 mm) of a nondenaturing micro 2D gel of human plasma proteins into 1 mm × 1 mm squares, in the area several proteins including high‐density lipoprotein (HDL) have been assigned by MALDI‐MS‐PMF 17, and the proteins in the 90 gel pieces were analyzed by quantitative LC‐MS/MS. The results showed that the method could provide native protein maps of more than 150 proteins and visualize the interactions of HDL apolipoproteins 1.

In this paper, we examined the performance of the method, i.e. nondenaturing 2DE followed by grid gel‐cutting and subsequent quantitative LC‐MS/MS, in the analysis of human cellular proteins. Soluble proteins of human bronchial smooth muscle cells (HBSMC) were separated by nondenaturing micro 2DE and a 30 mm × 40 mm area of the CBB‐stained slab gel (1.0 mm thick) was cut into 1.1 mm × 1.1 mm squares, then the proteins in the 972 gel pieces (squares) were applied to LC‐ion mobility separation‐enhanced MS/MS in data‐independent acquisition mode (HDMS^E^). Totally 4323 proteins were identified and the quantity distribution of each protein was reconstructed as a native protein map. These results for the first time visualized the distribution patterns of cellular proteins on a nondenaturing 2D gel, which would further provide information on their interactions. A method to evaluate the degree of similarity between protein maps was developed in order to examine the presence of protein complexes on the 2D gel and successfully applied to the 2328 protein maps that have three or more squares with the protein quantity data. The details are described separately 18.

## 2 Materials and methods

### 2.1 Materials

Human bronchial smooth muscle cells (HBSMC), which were isolated from human bronchi and supplied at secondary culture (or passage 1, P1), were from ScienCell Research Laboratories (Carlsbad, CA, USA) (Cat. No. 3400). SMCM medium, a basal medium supplemented with 2% v/v fetal bovine serum (FBS), 1% v/v smooth muscle cell growth supplement, 100 units/mL penicillin, and 100 μg/mL streptomycin, was also from ScienCell. The other reagents for cell culture and subculture were all from Hyclone, Thermo Fisher Scientific Inc. (Waltham, MA, USA). Human plasma from an apparently healthy individual (38 years, female) was collected as previously described 19. Agarose (Agarose IEF) and Pharmalyte pH 3–10 were both from GE Healthcare Life Sciences (Uppsala, Sweden). Tris(hydroxymethyl)aminomethane (Tris) and glycine, grade for electrophoresis, were from Bio‐Rad (Hercules, CA, USA). Low molecular weight (LMW) calibration kit for SDS electrophoresis (SDS not added) was from GE Healthcare (Little Chalfont, UK). Porcine sequencing‐grade modified trypsin was from Promega (Madison, WI, USA). MassPREP^TM^ Enolase Digestion Standard, prepared by digesting yeast enolase (SwissProt accession no. P00924), was from Waters (Milford, MA, USA). HPLC‐grade methanol and acetonitrile were from Fisher Scientific (Pittsburgh, PA, USA). All other chemicals, grades for electrophoresis, molecular biology, or MS, were purchased from Sigma (St. Louis, MO, USA). Water for all the solutions was prepared with a Milli‐Q Gradient A‐10 water system (Millipore, Bedford, MA, USA).

### 2.2 Cell culture and protein sample preparation

HBSMC were cultured in the SMCM medium (P1–P4) and then in a DMEM F‐12 medium supplemented with 10% v/v FBS, 100 units/mL penicillin, and 100 μg/mL streptomycin (P5–P7) and maintained at 37°C in humidified air containing 5% v/v CO_2_. Subculturing was conducted when the confluence reached to about 80–90% (about two days), with a split ratio of 1:3 (P1–P4) and 1:5 (P5–P7). Protein extraction was performed as follows. The P7 cells (grown to about 90% confluence) were washed with Dulbecco's phosphate‐buffered saline (DPBS) once and detached by incubating in a solution containing 0.05% w/v trypsin and 0.04 g/L EDTA for about 2 min at 37°C. The trypsinization was terminated by the addition of FBS (6% v/v final concentration) and the cells were collected by centrifugation at 160 × *g* for 5 min. The cell pellet was washed with ice‐cold DPBS for three times and ice‐cold GTE buffer (50 mM glucose, 25 mM Tris‐HCl, 10 mM EDTA, pH 7.4) for twice, and then added with a solution of 4 mM EDTA, 2 mM PMSF, 24% w/v glycerol (approximately equal volume of the cell pellet). The cells were resuspended by gentle stirring with a thin glass rod and immediately subjected to sonication using a cell disruption sonicator equipped with a φ3 titanium tip (VCX‐130 Sonics & Materials, Inc., Newtown, CT, USA). Keeping the sample tube soaked in ice water, the cells were sonicated for totally 60 s (10‐s pulse at 20% amplitude with 20‐s interval, six cycles). Cell debris was removed by centrifugation at 4°C for 10 min at 15 000 × *g*. The supernatant was collected and stored at –80°C in small aliquots. The protein concentration of the supernatant was estimated to be 15.4 μg/μL using a UV‐2600 UV‐VIS spectrophotometer (PerkinElmer, Waltham, MA, USA). Briefly, the supernatant was 100‐fold diluted with water, the absorbance of the solution was measured at 230, 280, and 320 nm, and the protein concentration was calculated with preset calibration parameters.

### 2.3 Nondenaturing micro 2DE

An aliquot of the sample solution (HBSMC supernatant fraction) was thawed and centrifuged just before 2DE at 4°C for 10 min at 15 000 × *g* to remove possible precipitate. Nondenaturing micro 2DE employing agarose IEF gels in the first dimension was run as previously described 15. Briefly, column agarose IEF gels with dimensions of 1.4 mm id × 47 mm length, which contained 1% w/v agarose and 5% v/v of Pharmalyte pH 3–10 (20‐fold dilution of the commercial solution), were used. Two hundred micrograms protein (13.0 μL of the sample solution) was loaded on an IEF gel from the cathodic end. The catholyte was 0.04 M NaOH–0.01 M NaCl and the anolyte was 0.01 M phosphoric acid, both precooled in ice water. The IEF apparatus was set in an ice‐water bath to cool the anolyte solution during focusing and the IEF gel column was immersed in the anolyte solution up to 41 mm from the gel bottom. IEF was run at 0.12 mA/gel constant current until the voltage increased to 300 V (about 23 min) and then continued at 300 V constant voltage for 25 min. The IEF gel was extruded by water pressure onto a plastic plate and the acidic end of the gel was cut off to give a final length of 37 mm, then the gel was transferred onto the top of a polyacrylamide micro slab gel (4.2–17.85% T linear gradient, 5% C, 42 mm high × 38 mm wide × 1 mm thick), where a 100‐μL aliquot of a 0.01 M Tris‐0.02 M glycine buffer (pH 9.0) was added beforehand. The second‐dimension electrophoresis was run using a 0.05 M Tris‐0.10 M glycine buffer (pH 9.0) at 10 mA/gel constant current and stopped 45 min after the line of BPB migrated out of the gel bottom (35 min) (totally 80 min). For estimation of the apparent p*I* and molecular masses of the separated proteins, coelectrophoresis of the HBSMC soluble proteins with human plasma and low‐molecular‐weight markers was performed. Briefly, a 10‐μL aliquot of the HBSMC soluble protein sample (*ca*. 160 μg) was mixed with a 1.0‐uL aliquot of human plasma sample (with 40% w/v sucrose) and loaded for the IEF run as described above. After IEF, the gel was extruded, trimmed, and set on the PAGE gel. An 18‐μL aliquot of a LMW marker solution, which contains six purified proteins with a total protein concentration 0.576 μg/μL and supplemented with 40% w/v sucrose, was then loaded above the IEF gel along its length, i.e. along the width of the slab PAGE gel. Nondenaturing micro PAGE was then run as described above.

### 2.4 CBB staining

The micro slab gel and the IEF gel were stained in 0.1% w/v CBB R‐250/50% v/v methanol/7% v/v acetic acid for 15 min, destained in 20% v/v methanol/7% v/v acetic acid for 2 h (at 25°C, one change) and kept in 7% v/v acetic acid. The color images of the CBB‐stained micro slab gel were scanned with an Epson V600 professional flat‐bed scanner (Seiko Epson, Suwa, Japan) at a 600 dpi resolution.

### 2.5 Grid gel‐cutting

**Figure 1:**
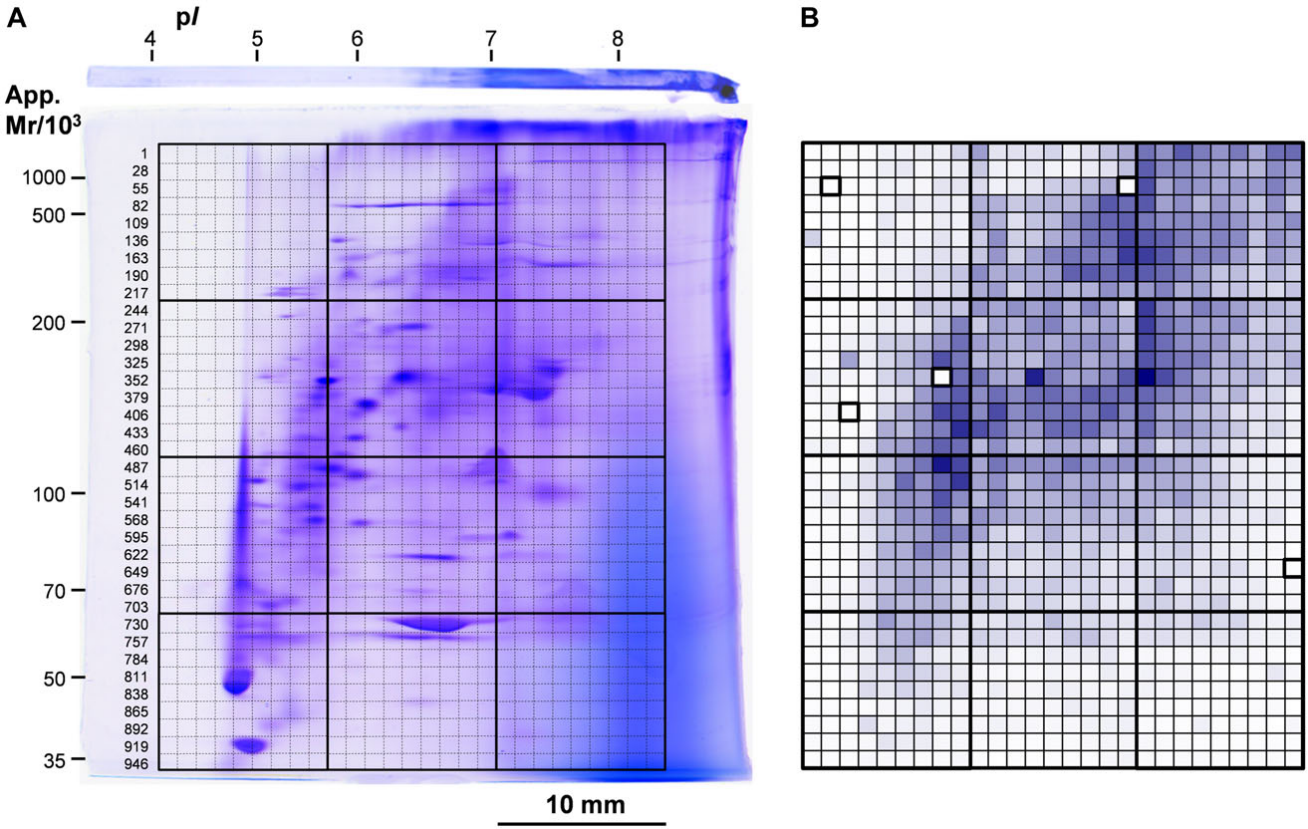
The nondenaturing micro 2D gel used for the analysis of HBSMC soluble proteins and the area of grid gel‐cutting. (A) The CBB‐stained micro 2D gel used for the analysis of HBSMC soluble proteins. A laboratory‐made device of grid gel‐cutting (Fig. 2) was used to cut the area of 30 mm × 40 mm into 972 gel pieces and each gel piece was numbered (numbers of the first square of each row were given at the left of the gel). (B) The distribution of protein quantity in the whole grid area was visualized using Excel macros as described in the text (Section 3.1). The positions of the five squares with no quantity data were shown as 100% transparent (white background) with dense closing lines. The estimation of the apparent p*I* and apparent mass was described in 18.

**Figure 2:**
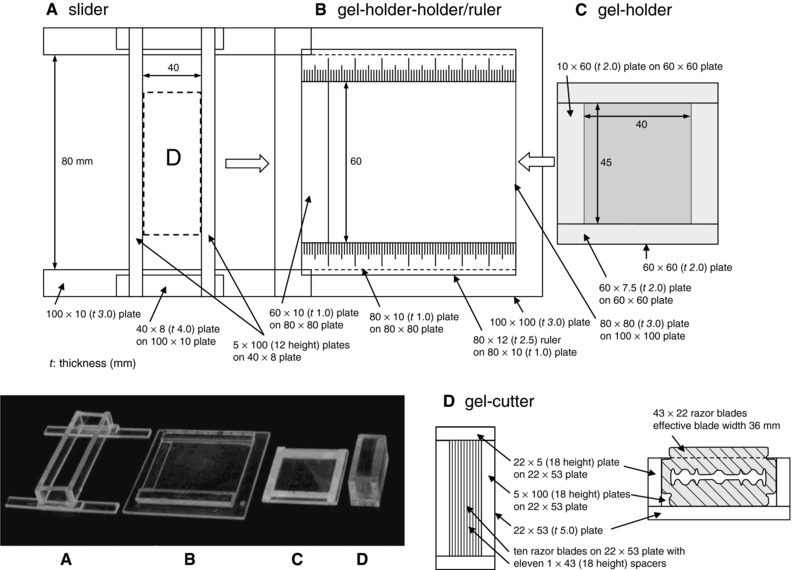
A laboratory‐made device for grid gel‐cutting. The CBB‐stained 2D gel (Fig. 1A) was set on the gel holder (part C) and the holder was set on the gel‐holder‐holder/ruler (part B), so as to the gel top of the micro 2D gel was set at the left. The slider (part A) was then slid into the holder/ruler to the premarked position and the 10‐blade cutter (part D) was placed into the slider and pressed down vertically. The cutter was then lifted up gently and the slider was moved horizontally to the right for 10 mm, and the second cutting was done. After the cutting was repeated for four times along the direction of second dimension separation (*cf*. Fig. 1A), the gel holder was turned 90° clockwise and cutting along the direction of first dimension separation was done for three times.

A 30 mm × 40 mm area of a CBB‐stained micro 2D gel (1.0 mm thick) was cut into 1.1 mm × 1.1 mm squares providing 27 × 36 gel pieces (972 squares) as shown in Fig. 1A. The grid gel‐cutting was performed using a laboratory‐made device shown in Fig. 2 as follows. The CBB‐stained gel was set on the gel holder (part C) and the holder was set on the gel‐holder‐holder/ruler (part B), so as to the gel top of the micro 2D gel was set at the left. The slider (part A) was then slid into the holder/ruler to the premarked position and the 10‐blade cutter (part D) was placed into the slider and pressed down vertically. The cutter was then lifted up gently and the slider was moved horizontally to the right for 10 mm, and the second cutting was done. After the cutting was repeated four times along the direction of second dimension separation (*cf*. Fig. 1A), the gel holder was turned 90° clockwise and cutting along the direction of first dimension separation was done three times. In this step, the power on the cutter was carefully adjusted so that the edges of the blades not reach to the surface of the gel holder (roughly cut up to 2/3 of the gel thickness), so that the gel squares stayed at their positions. Then the squares, altogether 972, were dissected one by one with the help of a razor knife and picked into microcentrifuge tube using a pair of sharp‐tip tweezers. The gel squares were stored in 7% v/v acetic acid at 4°C until further treatment (within 2.5 months).

### 2.6 Estimation of the apparent p*I* and molecular masses

The estimation of apparent p*I* and apparent masses of HBSMC proteins on the nondenaturing 2D gel was done using the results of coelectrophoresis of HBSMC soluble proteins, human plasma proteins, and LMW calibration proteins followed by the preparation of a standard curve for apparent mass estimation on protein maps. The details of the preparation of apparent mass standard curve was described elsewhere 18.

### 2.7 In‐gel digestion

The gel pieces were subjected to the procedures of destaining, reduction, alkylation, and in‐gel trypsin digestion, as described in detail in 19. The extracted peptides were dried by vacuum centrifugation and reconstituted with a 10 μL‐aliquot of 1% v/v formic acid‐2% v/v acetonitrile.

### 2.8 Nano‐ultra performance liquid chromatography (nano‐UPLC)

Prior to nano‐UPLC‐HDMS^E^ analysis, each tryptic peptide sample (10 μL) was added a 2 μL‐aliquot of the Enolase Digest Standard (240 fmol/μL) prepared in 1% v/v formic acid‐2% v/v acetonitrile. Nano‐LC separation of the peptides was performed with a nanoACQUITY system (Waters) equipped with a Symmetry 5 μm C_18_, 180 μm × 20 mm trap column and a nano‐ACQUITY 1.7 μm BEH130 C_18_, 75 μm × 100 mm analytical reverse phase column (both Waters). Mobile phase A was 0.1% v/v formic acid in water while mobile phase B was 0.1% formic acid in acetonitrile. Each sample, 5 μL full loop injection, was initially transferred with 99.5% mobile phase A‐0.5% mobile phase B to the trap column at a flow rate of 5 μL/min for 3 min. The peptides were then eluted from the trap column to the analytical column and separated at a flow rate of 300 nL/min with a gradient as follows: 2% B for 2 min, ramp to 50% B over 18 min, ramp to 85% B over 1 min, hold at 85% B for 5 min, then ramp to 2% B over 1 min. The column temperature was maintained at 35°C. The lock mass compound, leucine enkephalin (*m/z* 556.2771) (2 ng/μL) or [Glu1]‐Fibrinopeptide B (*m/z* 785.8427) (100 fmol/μL), was delivered at 0.05 μL/min to the reference sprayer of the NanoLockSpray source of the mass spectrometer *via* the sample pump of the MS apparatus.

### 2.9 Mass spectrometry

On‐line mass spectrometric measurement of the nano‐UPLC‐separated tryptic peptides was performed using a hybrid mass spectrometer coupling ion mobility separation (IMS) with Q‐TOF analyzer (Synapt G2‐S HDMS, Waters) and equipped with a nano‐ESI source. LC‐MS/MS data were collected in resolution, positive, and HDMS^E^ mode, i.e. ion mobility separation‐enhanced MS/MS in data‐independent acquisition mode, using settings that have been optimized based on the manufacturer recommendations. Briefly, the source settings included the capillary voltage of 3.5 kV, sampling cone of 50 V and the source temperature was 100°C; the analyzer settings for quadrupole profile set at manual with mass 1 as 400 (dwell time 25% and ramp time 25%), mass 2 as 500 (dwell time 25% and ramp time 25%), and mass 3 as 600. Nitrogen gas flow into the IMS cell at 90 mL/min and the helium gas into the helium cell (at the entry to the IMS cell) at 180 mL/min. IMS was performed before the collision, with trap wave velocity of 311 m/s and height of 4 V, 1000 μs delay after trap release, and IMS wave velocity of 652 m/s and height of 40 V. After IMS, collision was realized in the transfer cell with the energy held at 6 V for low energy scan and ramped from 20 to 45 V for the high energy scan with a collision gas flow (Ar) of 2.5 mL/min. Data were recorded alternating 0.8 s scans at low and elevated energy over a *m/z* 50–2000 range. The lock mass channel was sampled every 60 s.

### 2.10 Data processing, protein identification, and quantitation

The nano‐LC‐HDMS^E^ data were processed with ProteinLynx Global SERVER (PLGS) *ver*. 2.5.2 (Waters). Data were lock mass calibrated after acquisition. Peak processing parameters were the low energy threshold 20 counts, high energy threshold 10 counts, and intensity threshold 500 counts. Database searching were performed using the following parameters: database, UniProtKB homo sapiens complete proteome dataset (canonical sequences only, 20 251 entries, 2013‐05‐29); peptide and fragment tolerance, both automatic (typically <10 ppm and <20 ppm, respectively 20); maximum of missed trypsin cleavage, 1; maximum protein mass, 600 kDa; fixed modification, carbamidomethylation at Cys; variable modification, oxidation at Met; false‐positive rate (protein level), 4%. The criteria of protein identification were set as; at least two peptide matches per protein, at least three fragment ion matches per peptide, at least seven fragment ion matches per protein, and protein score above 100. Protein quantities were calculated by PLGS referring to the quantity of the internal standard (tryptic peptides of ENO1_YEAST). Calculated masses of the proteins assigned by nano‐LC‐HDMS^E^ were calculated using a laboratory‐made Visual Basic program after searching the information of each protein in Protein Knowledgebase (UniProtKB) and getting the protein chain sequence (without signal peptide and propeptide).

## 3 Results and discussion

### 3.1 HBSMC soluble proteins analyzed by the combined method of nondenaturing micro 2DE, grid gel‐cutting, and quantitative LC‐MS/MS (HDMS^E^)

Previously, we reported on a new method of protein analysis that combined nondenaturing micro 2DE, grid gel‐cutting, and quantitative LC‐MS/MS (in data‐independent acquisition mode, or MS^E^) 1. A 5 mm × 18 mm gel area of a CBB‐stained micro 2DE pattern of human plasma was cut into 1 mm × 1 mm squares, each of the 90 gel pieces (1 mm^3^) was subjected to in‐gel digestion and LC‐MS/MS, and the quantity distributions of the 154 assigned proteins were reconstructed to “native protein maps” that visualized the localization of proteins and provided information on lipoprotein interactions. We attempted to examine the performance of this approach in the analysis of human cellular proteins. Human bronchial smooth muscle cells were selected since our laboratory has research groups working on the molecular mechanisms of asthma and future collaboration was expected. The nondenaturing 2DE conditions that have been developed for the analysis of *E. coli* proteins 15 were employed for the analysis of HBSMC soluble proteins, except only “long‐run” conditions of second dimension run were used to assist size‐separation of proteins in the gradient gels, at the cost of restricting the apparent mass range of proteins from *ca*. 3000 to 30 kDa. The CBB‐stained nondenaturing micro 2D gel and the area of grid gel‐cutting (30 mm × 40 mm) were shown in Fig. 1A. The method of grid gel‐cutting was used expecting the following three merits; (1) avoid missing the assignment of proteins in the faintly stained gel area, (2) standardize the conditions of the gel‐piece treatment including destaining, reducing and alkylation, in‐gel trypsin digestion, and peptide extraction, (3) enable the quantity mapping of the proteins distributed in the grid area. We employed LC‐MS/MS in HDMS^E^ mode 21, 22 rather than MS^E^ mode 23, 24 because of the higher sensitivity in detecting low‐abundant proteins. The analysis of each of the gel pieces by nano‐UPLC‐HDMS^E^ provided the assignment of 2–280 proteins (average about 80), together with the information on their quantity. Totally 4323 protein species were assigned in 967 gel pieces out of the 972 and the results were shown in Supporting Information Table 1. Two gel pieces were not analyzed since we judged that they were contaminated during the manual procedures of peptide extraction and the MS data of three gel pieces were not acquired, two by momentary malfunction of the electrospray emitter and one by a mistake in data handling. The protein quantities measured in each gel piece (square) were summed up and the distribution of protein quantity in the grid area was visualized using the following procedure with the aid of Excel macros. (1) An Excel worksheet that contains 967 rows with the columns of “square number” and “quantity sum” was prepared and the data were sorted in the order of quantity. The way of square numbering was shown in Fig. 1A. (2) The protein quantity value of each square was converted to a percent value, setting the highest quantity among the 967 squares, i.e. the quantity of square 370, to be 100%, making a new column of “percent quantity.” (3) A grid of 27 columns × 36 rows was drawn and each numbered square was painted with a color (blue), the transparency in % of which was determined by calculating [100% ‐ “percent quantity” of the square]. The results are shown in Fig. 1B. The positions of the five squares with no quantity data were shown as 100% transparent (white background) with dense closing lines. The pattern generally reproduced the CBB stained pattern of the grid area, but the spot resolution was much lower as it is inevitable for an image of 27 × 36 pixels.

### 3.2 Preparation of native protein maps of LC‐MS/MS‐assigned HBSMC soluble proteins

**Figure 3:**
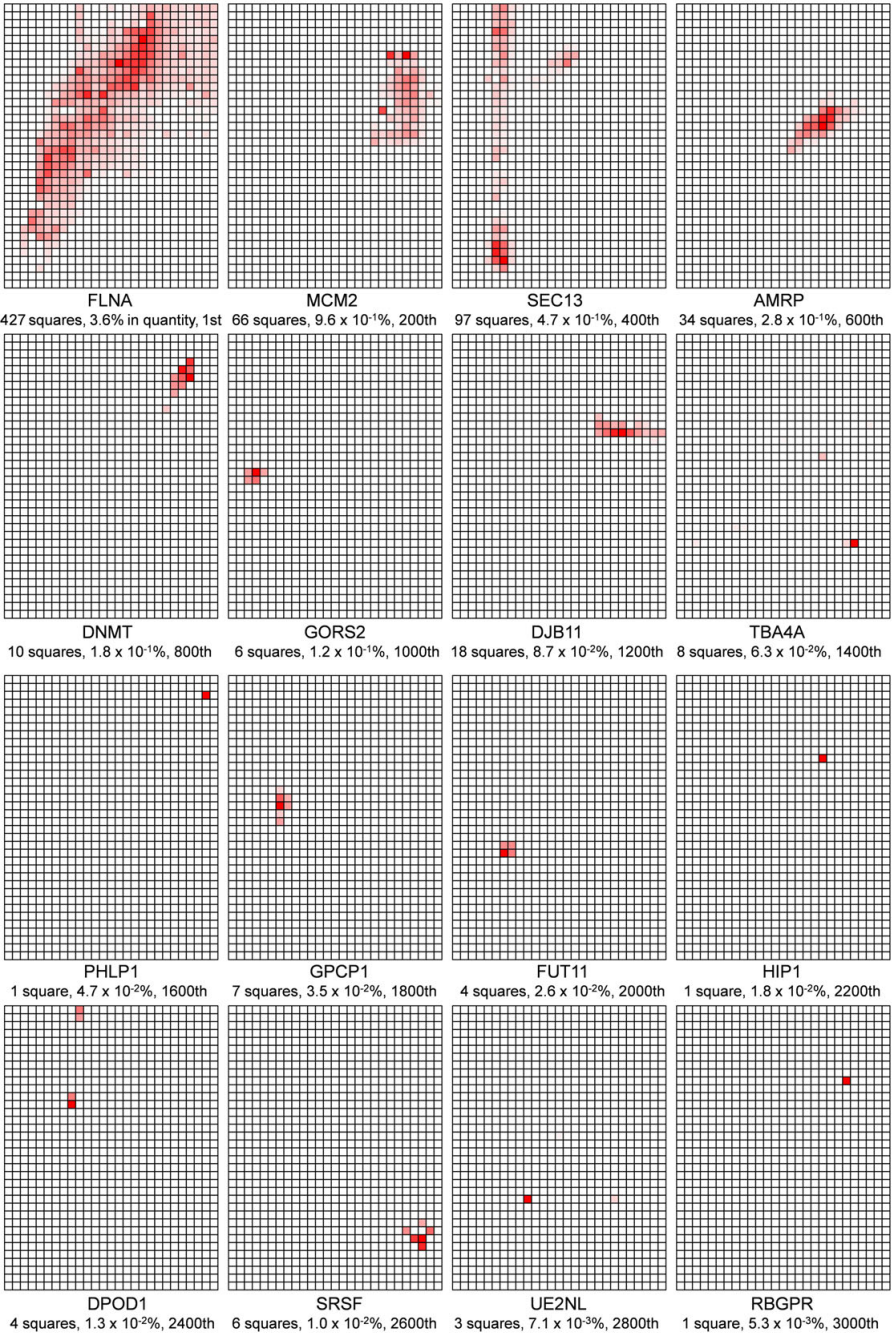
Sixteen examples of the native protein maps of HBSMC soluble proteins. Each map was added the description on UniProt protein entry name (without “_HUMAN”), number of squares detected, percent abundance against the total protein quantity within the grid area, and the rank of the protein in the quantity list sorted in descending order.

**Figure 4:**
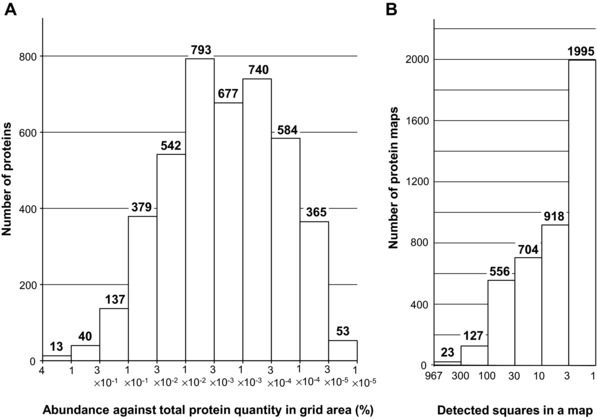
A frequency histogram of the identified HBSMC proteins that have a particular percent abundance against the total protein quantity within the grid area (A) and a frequency histogram of the number of protein map that showed a particular number of detected square(s) in the map (B).

One of the aims of grid gel‐cutting was to reconstruct the quantity distribution patterns (native protein maps) of the assigned proteins. The procedures previously used to draw maps of 5 × 18 squares for the analysis of human plasma HDL and its apolipoproteins 1 were extended to draw maps of 27 × 36 squares for the analysis of HBSMC soluble proteins using Excel macros; (i) put a tag of the square number to all the data rows that include information on protein name, protein quantity, etc. (about 80 000 rows), (ii) collected all data in one worksheet and sorted them by “protein entry name” as the first priority and “quantity in ng” as the second priority to align each assigned protein in the order of quantity (Supporting Information Table 1), (iii) copied the data of each assigned protein (maximum 967 lines with different square numbers) to a new worksheet, (iv) converted the values in the column of “quantity in ng” in each protein's worksheet to percent values, setting the highest quantity to be 100%, making a new column of “percent quantity,” (v) drew 27 (columns) × 36 (rows) squares to form a grid on each worksheet and to paint each square with a color, the transparency in % of the color (red) was determined by calculating [100% ‐ “percent quantity” of the square]. Using the procedure, the distribution of a protein, in terms of its relative quantity within the grid area, was reconstructed as a color density pattern (a native protein map). Figure 3 shows 16 examples of protein maps out of the total 4323 maps, demonstrating that each protein has characteristic quantity distribution that is differentiated from others by many features, such as the position of quantity peak square in p*I* and apparent mass axes, number of detected squares, degree of concentration (focused or dispersed), shape of the colored square group (lengths in horizontal and vertical directions), angle of the longest axis, etc. Each map in Fig. 3 was added the description on protein entry name (without “_HUMAN”), number of squares detected, percent abundance against the total protein quantity within the grid area, and the rank of the protein in the quantity list sorted in descending order. Since each protein map is composed from the squares that represent only the relative quantity against the highest quantity, the value of percent abundance must be always accompanied with the map, when the results of nano‐UPLC‐HDMS^E^ are summarized. Although each protein map should include specific information on the individual protein, it is difficult to show the 4323 protein maps one by one, so some of the features of the maps were extracted as shown in Fig. 4. Figure 4A is a frequency histogram of the identified HBSMC proteins that have a particular percent abundance against the total protein quantity within the grid area. The percent abundance ranged from 3.6% to 1 × 10^–5^% of the total protein quantity in the grid area. It is noteworthy that the range of the percent abundance in one square was much narrower. For example, in square 370, in which the largest protein quantity was detected, the percent abundance of the 164 protein species spans across only three orders of magnitude, ranging from 0.031% to 4 × 10^–5^%. These results suggest that the method of grid gel‐cutting and mapping not only served to visualize the distribution of proteins, but also worked to expand the dynamic range of protein quantitation. Figure 4B is a frequency histogram of the number of protein map that showed a particular number of detected squares in the map. About 46 percent of the proteins were detected in 1 or 2 squares, about 50% were detected in 3–99 squares, and about 4% of the proteins were detected in 100 or more squares. The last type of proteins generally showed wide distribution on their map both in p*I* direction and in apparent mass direction, as typically shown in Fig. 3 FLNA (filamin A). The proteins detected in 100 or more proteins include structural constituents of cytoskeleton (vimentin, actins, tubulins, etc.), chaperons (78 kDa glucose regulated protein, heat shock cognate 71 kDa protein, 14‐3‐3 proteins, etc.), and glycolysis enzymes (glyceraldehyde‐3‐phosphate dehydrogenase, pyruvate kinase PKM, L lactate dehydrogenase A, and B chains, etc.). These proteins might have the ability to interact with various proteins and their wide distribution might reflect the heterogeneity in the apparent p*I* and apparent mass of the complexes. Although some keratins were also detected in above 100 squares, we judged that they might have contaminated in the manual steps of gel preparation, electrophoresis, grid‐cutting, etc., since they were randomly detected throughout the gel area at similar quantity levels.

### 3.3 Peak positions of focused HBSMC soluble proteins

**Figure 5:**
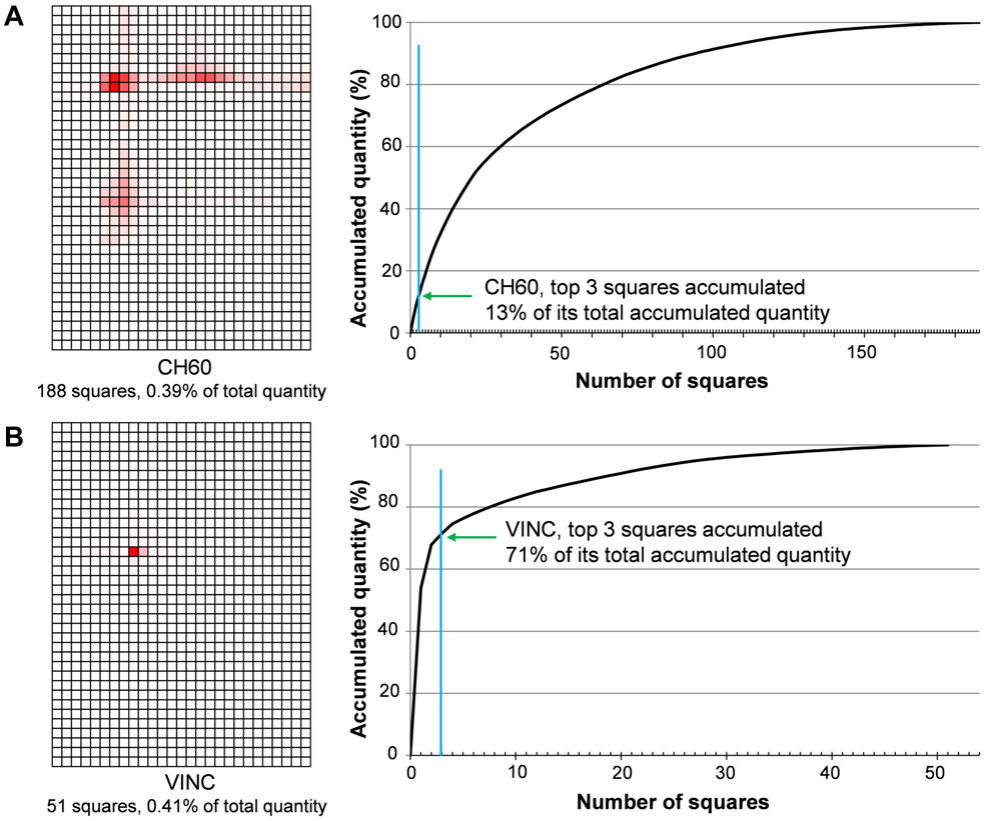
Two examples of protein map with different quantity distributions. (A) Mitochondrial 60 kDa heat shock protein (CH60) was detected in 188 squares and the sum of the quantity of its top‐three squares accounted for 13% of the total quantity. (B) Vinculin (VINC) was detected in 51 squares and the sum of the quantity of its top‐three squares accounted for 71% of the total quantity.

**Figure 6:**
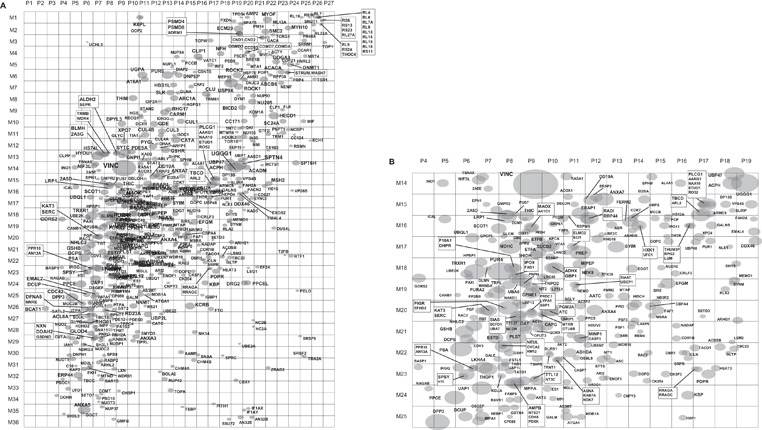
The peak positions of relatively focused proteins. The criteria to select the relatively focused proteins and the procedure to draw the peak positions were described in detail in Section 3.3. (A) Peak positions of the 565 selected proteins. The squares were indicated by P1–P27 in the p*I* direction and M1 to M36 in the apparent mass direction. When the peak positions were overlapped, the entry names of the proteins were shown in box frames. (B) Expanded view of the region of P4–P19 and M14–M25 to show the protein entry names difficult to read in (A).

In the course of summarizing the features of the protein maps, we noticed that some proteins had dispersed quantity distribution and their quantity peak positions were difficult to define, suggesting that they might form protein complexes with other proteins. However, a large number of proteins had rather focused quantity distribution. Figure 5 shows two examples of protein maps with different quantity distributions. Mitochondrial 60 kDa heat shock protein (CH60, Fig. 5A) is a chaperon protein and was detected in 188 squares. The sum of the quantity of its top‐three squares accounted for 13% of the total quantity in the 188 squares and the dispersed but unique distribution pattern specified the map of the protein. In contrast, vinculin, which is known to have multiple functions including actin filament binding, was detected in 51 squares (VINC, Fig. 5B) and the sum of the quantity of its top‐three squares accounted for 71% of the total quantity. Then we decided to define the quantity peak positions of relatively focused proteins and summarize them as a map. The criteria of protein maps for the calculation of quantity peak positions were as follows and the selection was done by preparing Excel macros; (i) the protein was detected in five or more squares (1783 maps), (ii) the quantity of top‐three squares of the protein accounted for 40% or more of the total quantity (941 maps out of the 1783), and (iii) the top‐three squares were adjacent, e.g. each square shared at least one of the corners with one of the other two squares (565 maps out of the 941). The peak positions of the 565 proteins were illustrated as shown in Fig. 6 using Excel macros employing the following procedure; (i) the peak position (*x*_peak_, *y*_peak_) of a protein was calculated as below, assuming the protein quantity in a square is concentrated at the center of the square;

x peak =q1x1+q2x2+q3x3/(q1+q2+q3),y peak =q1y1+q2y2+q3y3/(q1+q2+q3),

where *q_1_*, *q_2_*, and *q_3_* are the quantities of the protein in the top‐3 squares, *x_1_*, *x_2_*, and *x_3_* are the positions (integers within 1–27) of the three squares in p*I* axis, *y_1_*, *y_2_*, and *y_3_* are the positions (integers within 1–36) in apparent mass axis, respectively, (ii) the spot shape was assumed to be an ellipse with a ratio of major (*x*) axis to minor (*y*) axis to be 3:2, (iii) the length of the major axis was decided to be proportional to the cube root of the sum of the top‐3 protein quantity. The range of the top‐3 quantity of the 565 proteins was from 2.9 × 10^–1^% to 1.9 × 10^–4^% of total protein quantity in the 30 mm × 40 mm gel area, so in Fig. 6 the length of the major axis of the biggest spot is about tenfold larger than the smallest spot. The data used to prepare Fig. 6A and B are provided as Supporting Information Table 2. It must be noted that the maps shown as Fig. 6A and B were prepared to illustrate the peak positions and relative quantities of the proteins defined above and not directly related to the protein distributions in native protein maps. Actual quantity distribution map of each protein can be reproduced using data in Supporting Information Table 1.

### 3.4 Features and performance of the employed method in proteomic analysis of cellular proteins

Since the method employed here, the combined method of nondenaturing micro 2DE, grid gel‐cutting, and quantitative LC‐MS/MS in HDMS^E^ mode, was for the first time applied for the analysis of cellular proteins, we summarized the features and performance of the method in this section.

Nondenaturing 2DE: Nondenaturing 2DE has been used to separate proteins retaining their functions, such as enzyme activities and protein–protein interactions, then the proteins fixed and stained on the 2D gel were excised and digested for peptide analysis by MS 15, 16. In contrast, denaturing 2DE has been used to separate single‐chained polypeptides 2 and the polypeptides (preferably a polypeptide) in a gel piece were digested. Further, cellular proteins were directly digested without employing the step of protein separation 3, 4, 5. Although all these methods aim at the proteomic analysis of cellular proteins, the content of the information provided by the methods would differ according to the differences in the approach, such as (i) information on intact proteins and their interactions, (ii) information on single‐chained polypeptides and their modifications, and (iii) total analysis of peptides and thorough identification of proteins. Therefore, these methods should be complementary in the analysis of cellular proteins and the current method would provide information on cellular proteins and protein interactions.

Agarose IEF gel and micro gel format: We replaced polyacrylamide IEF gels with agarose IEF gels in order to separate large protein complexes 25 and the micro gel format have been employed in order to minimize the time of separation of native proteins (*ca*. 130 min total 2DE run time). Also, the micro gel format facilitated the grid gel‐cutting and the mass spectrometric analysis of the gel pieces.

Grid‐cutting of the gel: Previously, we identified *E. coli* soluble proteins excising the stained spots on a nondenaturing 2D gel and analyzing the in‐gel digested peptides with MALDI‐MS‐PMF 15. However, we noticed that the sensitivity of the apparatus of quantitative LC‐MS/MS in protein identification exceeded the sensitivity of conventional protein staining techniques and thus employed grid gel‐cutting instead of excising stained gel pieces 1. The method of grid gel‐cutting worked as an interface step between an analog method (2DE) and a digital data treatment. The information of protein distribution and quantity that have been buried in a nondenaturing 2D gel could be digitally reproduced as 4323 protein maps. Since the quantity range of proteins in one gel piece should be much narrower than that of whole cellular proteins, we expect the proteins in the gel pieces could be analyzed by mass spectrometric apparatus with narrower dynamic range but higher sensitivity than the one used in this work, such as Orbitrap mass spectrometers, to obtain more protein maps of low‐abundant proteins.

Information included in each protein map: Each protein map reflects the behavior of the specific protein during its migration in the agarose IEF gel and then in the polyacrylamide gradient gel. The quantity distribution of the 4323 proteins in the gel area of 30 mm × 40 mm can be reconstructed and visualized from the data provided as Supporting Information Table 1 and the information obtained from each protein map was in part summarized in Sections 3.1–3.3. Further, each protein map included information related to its complex formation. As shown in Fig. 1A, the proteins in the grid area showed apparent masses ranging from about 30 kDa to more than 1000 kDa, although the average calculated mass of the 4323 proteins identified was about 62 kDa. Soluble proteins of *E. coli* analyzed by nondenaturing 2DE followed by MALDI‐MS‐PMF also showed much higher apparent mass values than their calculated ones, and the reason was attributed to the formation of protein complexes, homo‐oligomers, and hetero‐oligomers of proteins 16. Therefore, examination of each protein map would provide information on the possibility of the formation of protein complexes. Also, the quantity peak position of a protein on the gel could be excised and subjected to SDS‐PAGE (three‐dimensional electrophoresis, 3DE), in order to obtain detailed information on protein interactions 16, 26. Each protein map would facilitate to purify a specific protein of interest by excising the corresponding gel area just after nondenaturing 2DE, as a micropreparative technique of proteins retaining their physiological functions. The detection of protein physiological functions using nondenaturing 2DE have been reported on protein complexes in human plasma 25, enzymes in mouse liver soluble protein fraction 27, and enzymes in *E. coli* soluble protein fraction 15. Also, further purification of the specific protein would be done by 3DE, as have been reported on human plasma proteins 26 and on *E. coli* proteins 16.

Comparisons of protein maps: Comparisons of the native protein maps would also provide information on the differences in the characteristics of the proteins. As described in Section 3.2, the proteins widely detected over the gel area might have the ability to interact with various proteins and their wide distribution might reflect the heterogeneity in the apparent p*I* and apparent mass of the complexes. In contrast, the rather concentrated proteins described in Section 3.3 and shown in Fig. 6 might represent proteins that do not have general ability of protein interaction. However, the possibility of interactions between several protein species must be judged by one‐by‐one comparisons between protein maps. The proteins that comprise a protein complex would tend to migrate together under the conditions of nondenaturing 2DE and when the protein complex did not dissociate during the whole steps of 2DE, the component proteins would show similar quantity distribution patterns (native protein maps). Therefore, the information of such protein complexes would be obtained by examining the similarity of native protein maps. As for the comparisons between protein maps, we developed Excel macros to extract protein pairs with similar protein distribution and examined whether each protein pair was described to form a complex in a database (UniProtKB). The details of the results are described separately 18.

Thorough analysis of cellular proteins: The method of direct protein digestion without the step of protein separation 3, 4, 5 would be the most suited for the thorough identification of cellular proteins. We identified the HBSMC 4323 protein species from the 30 mm × 40 mm gel area and the number is compatible with those obtained by direct digestion of cellular proteins followed by LC‐MS/MS that identified about 10 000 proteins 6, 7. However, the results of protein analysis on low‐apparent mass proteins (smaller than *ca*. 30 kDa), on the proteins stayed at the 2D gel‐top, at the 2D gel basic end, in the agarose IEF gel (*cf*. Fig. 1A), and in the fraction precipitated in the step of centrifugation, were not described in this paper since we focused on the results of protein map preparation from the 30 mm × 40 mm gel area. Aiming at more thorough analysis of cellular proteins, we are analyzing the proteins in the fractions mentioned above and the results will be described elsewhere. The fractionation of proteins and the analysis of all the fractions would require much labor than the method of direct protein digestion. However, we expect that proteins in each fraction would have properties characteristic to the fraction, such as membrane protein–lipid complexes that could not migrate into the agarose IEF gel or high‐molecular‐mass protein complex that could migrate into the agarose IEF gel, but could not migrate in the polyacrylamide gradient gel. In order to precisely compare the performance of the current method with the method of direct protein digestion on the number of identified proteins, proteins in all the fractions we obtained should be analyzed using an Orbitrap MS apparatus, which have been employed in direct protein digestion identifying 10 000 proteins 6, 7.

## 4 Concluding remarks

The combined method of nondenaturing micro 2DE, grid gel‐cutting, and quantitative LC‐MS/MS provided the identification of 4323 HBSMC proteins from a 2D gel area of 30 mm × 40 mm and the treatment of the MS/MS results using Excel macros provided for each of the identified protein a distribution map (a native protein map) with the quantity information. These results for the first time visualized the distribution patterns of cellular proteins on a nondenaturing 2D gel. Each protein map showed where a specific protein reside on the nondenaturing 2D gel and the information would facilitate to purify a protein of interest using nondenaturing 2DE and to examine its physiological functions and interactions with itself or with other proteins. Each protein map showed the characteristic distribution of the protein, reflecting the degree of concentration in p*I* and apparent mass axes. The comparisons between protein maps would help to analyze protein complexes separated on nondenaturing 2D gels. Although we concentrated to analyze in detail one nondenaturing 2DE gel in this paper, the performance of the method will be further examined including the gel‐to‐gel and sample‐to‐sample reproducibility.

*The mass spectrometry proteomic data have been deposited to the ProteomeXchange Consortium* (http://www.proteomexchange.org) *via the PRIDE partner repository [* 28 *] with the dataset identifier PXD001471. The authors would like to thank the PRIDE team for their great help with the data deposition process*.

*The authors have declared no conflict of interest*.

## Supporting information

Table S1.

Table S2.
